# Effects of synthetic peptide RP557 and its origin, LL-37, on carbapenem-resistant *Pseudomonas aeruginosa*

**DOI:** 10.1128/spectrum.00430-23

**Published:** 2023-08-09

**Authors:** Yun-Qi Song, Su Min Kyung, Suji Kim, Gun Kim, So Yeong Lee, Han Sang Yoo

**Affiliations:** 1 Department of Infectious Disease, Seoul National University, Seoul, Republic of Korea; 2 Research Institute for Veterinary Science, Seoul National University, Seoul, Republic of Korea; 3 Laboratory of Veterinary Pharmacology, Seoul National University, Seoul, Republic of Korea; The Pennsylvania State University, University Park, Pennsylvania, USA

**Keywords:** antimicrobial peptide, carbapenem resistance, biofilm, *Pseudomonas aeruginosa*

## Abstract

*Pseudomonas aeruginosa* is a common bacterium in nosocomial infection. The biofilm-forming ability and antimicrobial resistance make *P. aeruginosa* biofilm infection refractory to patients requiring hospitalization, especially patients in the intensive care unit. Therefore, many alternative compounds have been developed. A newly synthesized peptide, RP557, derived from human cathelicidin LL-37, was evaluated for its antimicrobial and antibiofilm effect toward carbapenem-resistant *P. aeruginosa* (CRPA). The results showed that regardless of the resistance to carbapenems, the minimal inhibition concentrations of RP557 and LL-37 against *P. aeruginosa* were 32 µg/mL and 256 µg/mL, respectively. Both RP557 and LL-37 significantly reduced the *P. aeruginosa* biofilm mass at subMICs, while subMICs of carbapenems induced biofilm formation. RP557 could also remove approximately 50% of the mature biofilm at a concentration of 64 µg/mL, while 256 µg/mL LL-37 was needed to remove it. A quarter MIC of RP557 and LL-37 was used together with carbapenems (ertapenem, imipenem, and meropenem). The results show that both RP-557 and LL-37 might increase the susceptibility to CRPA by 4–16 times. Significant gene expression level changes were observed in RP557- or LL-37-treated CRPA. Confocal images showed that biofilm structures and biofilm cell viability were significantly reduced in the LL-37- or RP557-treated groups. Therefore, RP557 and its structural origin, LL-37, could be potential treatments for carbapenem-resistant *P. aeruginosa* infection, especially for chronic biofilm infection.

**IMPORTANCE:**

*Pseudomonas aeruginosa* is one of the major pathogens of nosocomial infection. Combined its biofilm-forming ability with carbapenem-resistance, it is hard to handle *P. aeruginosa* infection, especially for patients requiring hospitalization. Antimicrobial peptide is a type of potential compound for bacterial infection treatment. Among these, RP557 was found effective in inhibiting biofilm previously. By assessing its effect on both carbapenem-resistant *P. aeruginosa* planktonic cells and biofilm, our results offered a potential treatment for carbapenem-resistant *P. aeruginosa* infection. It could be helpful to treat severe nosocomial infection related to carbapenem-resistant bacteria and increase the patients’ survival rate.

## INTRODUCTION

*Pseudomonas aeruginosa* is a ubiquitous opportunistic pathogen that often causes nosocomial infection, with approximately 16.2% of intensive care unit (ICU) infections being caused by *P. aeruginosa* (1). As typical biofilm-forming bacteria, *P. aeruginosa* biofilms often cause wound infections, catheter-related infections, and chronic lung infections in cystic fibrosis patients (2). *P. aeruginosa* biofilm infection is hard to cure because biofilms are usually more resistant to antibiotics than the planktonic form. The antimicrobial resistance mechanism of biofilms can be divided into two parts. First, the anionic components of the biofilm can reduce the penetration of cationic antibiotics, thus protecting bacterial cells from antibiotics. Second, there are metabolic differences between biofilm cells. At the stalk part of the biofilm, some cells are metabolically inactive, so they are resistant to antibiotics that act during the bacterial growth phase (3). In addition to its intrinsic resistance to β-lactam antibiotics, the development of resistance to carbapenems makes *P. aeruginosa* infection harder to cure. The carbapenem-resistance mechanism of *P. aeruginosa* can be divided into four types: overexpression of efflux pumps, porin mutation, derepression of intrinsic β-lactamase, and production of carbapenemase. Among them, mutations in porins are the main mechanism of carbapenem resistance in *P. aeruginosa*, especially to imipenem (4 – 6). Combined with its strong biofilm-forming ability, carbapenem-resistant *P. aeruginosa* (CRPA) results in higher mortality than carbapenem-resistant Enterobacteriaceae (7). Thus, new therapeutic compounds should be developed for treating CRPA infection.

Antimicrobial peptides (AMPs) are peptides that display promising antimicrobial effects. They can be naturally produced by plants, fungi, and bacteria. Most AMPs are positively charged and achieve their antimicrobial effect by disrupting microorganism cell membranes through electrostatic interactions with anionic phospholipids, which further leads to cell death (8, 9). The antimicrobial effect of an AMP can be enhanced by adjusting its structure, which provides a basis for the synthesis of new AMPs. LL-37 is the only cathelicidin secreted in humans and is often found in mucosal surfaces (10). LL-37 has been shown to have antimicrobial effects by disrupting the lipid bilayer. However, compared to its moderate antimicrobial effect on planktonic cells, LL-37 has an outstanding effect on biofilms that reduces biofilm formation at subMICs (11, 12). Thus, many antimicrobial peptides have been designed based on LL-37. RP557 is a novel synthetic peptide derived from LL-37 and D2A21, a synthetic antimicrobial peptide derived from cecropin (13). It has a broad-spectrum antibacterial and antibiofilm effect against common Gram-negative and Gram-positive bacteria and even fungi. RP557 is reported to have low cytotoxicity and to be less likely to develop resistance to *P. aeruginosa* and *Staphylococcus aureus* than gentamicin and clindamycin (13). In another study, RP557 was found to be effective in the biofilm of multidrug-resistant *Mycobacterium abscessus* and can increase susceptibility to other antibiotics when coadministered (14).

In the present work, we evaluated the antimicrobial effect of RP557 and LL-37 on CRPA planktonic cells and biofilms. We also evaluated the carbapenem effect on CRPA biofilms and found that biofilms may contribute to carbapenem resistance. Then, the effect of RP557 and LL-37 on mature biofilms was presented as well. The synergetic effect between RP557/LL-37 and carbapenems on CRPA showed that combining medications can dramatically increase bacterial susceptibility to carbapenems. Then, the antibiofilm mechanism of RP557 and LL-37 was evaluated by quantitative PCR, and the effect on biofilm structure was visualized by confocal laser scanning microscopy.

## MATERIALS AND METHODS

### Bacteria strains and culture condition

*Pseudomonas aeruginosa* reference strain ATCC 27853 was obtained from the American Type Culture Collection (Manassas, VA, USA). Carbapenem-resistant *P. aeruginosa* strain 16079 was obtained from the Korean National Culture Collection for Pathogens (NCCP, Cheongju, South Korea). Other CRPA strains were sampled from companion animals by veterinarians and preserved in the infectious disease laboratory (Seoul National University, Seoul, South Korea). Strains D7, D16, and D26 were isolated from companion dogs with otitis. Strain D25 was isolated from companion dogs with pyoderma (15), and strain B4 was isolated from the feces of companion dogs. Bacteria were maintained on tryptic soy agar during the experimental process (BD, Franklin Lakes, USA) and enriched in tryptic soy broth (BD, Franklin Lakes, USA).

### Antibiotics and antimicrobial peptides

Meropenem and imipenem were purchased from Sigma Aldrich (Saint Louis, MO, USA). Ertapenem was purchased from European Pharmacopoeia Reference Standard (Strasbourg, France). Antimicrobial peptides LL-37 (LLGDFFRKSKEKIGKEFKRIVQRIKDFLRNLVPRTES) and RP557 (RFCWKVCYKGICFKKCK) were synthesized by GL Biochem (Shanghai, China) and purified to 95% by high-performance liquid chromatography. Lyophilized peptide was stored at −20°C until use.

### Minimal inhibitory concentration assays

The MIC assay was performed following the guidelines from the Clinical and Laboratory Standards Institute with slight modifications (16). Briefly, *P. aeruginosa* was incubated at 37°C overnight and diluted in cation-adjusted Muller-Hinton broth (CAMHB) to approximately 1 × 10^6^ CFU/mL. Bacteria were incubated with 2-fold diluted carbapenems or peptides in 96-well plates (Greiner Bio-One, Frickenhausen, Germany) at 37°C overnight. After incubation, the final OD_600_ was measured using the VersaMax absorbance microplate reader (Molecular Devices, CA, USA), and the minimal inhibition concentration was determined by the concentration of wells at which no bacterial growth was detected. The assay was performed in triplicate, with each sample performed in triplicate wells.

### Biofilm inhibition assay

Biofilm inhibition assays were performed using the microtiter plate method previously described with slight modifications (17). Briefly, *P. aeruginosa* was prepared and diluted using CAMHB to 1 × 10^6^ CFU/mL. Twofold diluted solutions of LL-37 (512 to 1 µg/mL) and RP557 (128 to 0.25 µg/mL) were prepared. Ertapenem, imipenem (512 to 1 µg/mL), and meropenem (256 to 0.5 µg/mL) were prepared on 96-well U-bottom microtiter plates. Then, an aliquot of 50 µL of bacterial suspension was inoculated into each well. Plates were covered and sealed and incubated at 37°C for 24 h without agitation. Biofilm mass was assessed using the crystal violet staining method. Briefly, after incubation, the bacterial suspension was discarded, and the wells were washed with PBS buffer. Then, each well was stained with 120 µL 0.1% crystal violet dye for 30 min. After staining, the dye was discarded, and excess dye was washed out using distilled water. Then, 120 µL 100% ethanol was added to dissolve the dye. After 20 min of incubation, the OD_570_ was measured. Biofilm mass was normalized by comparing it to the growth control group and represented as a percentage. The concentration at which biofilm formation is inhibited by over 90% is considered as the minimal biofilm inhibition concentration (MBIC). All assays were performed in triplicate, with each sample performed in triplicate wells.

### Biofilm removal assay

Overnight cultures of *P. aeruginosa* were prepared and diluted in CAMHB to approximately 1 × 10^6^ CFU/mL. An aliquot of 100 µL of bacterial suspension was inoculated into each well and incubated at 37°C for 24 h to establish a mature biofilm. Then, the suspension was discarded, and each well was washed three times using PBS to wash out unattached cells. LL-37 and RP557 were dissolved in CAMHB at concentrations ranging from 0.25 μg/mL to 256 μg/mL. Each well was loaded with peptide solution and further incubated at 37°C for 24 h without agitation. The remaining biofilm mass was evaluated by 0.1% crystal violet staining followed by measurement of optical absorbance at 570 nm. Biofilm mass was normalized by comparison to the growth control group and represented as a percentage. The concentration at which biofilm is removed by over 90% is considered as the minimal biofilm inhibition concentration (MBEC). The experiments were performed in triplicate, and each sample was analyzed in triplicate wells.

### Evaluation of the synergetic effect between antimicrobial peptides and carbapenems

To assess the combined effect of antimicrobial peptides and carbapenems, 25 µL of peptide solution at the MIC and 25 µL of carbapenem solution with concentrations ranging from 1 μg/mL to 2048 μg/mL were loaded onto 96-well plates. Overnight cultures of *P. aeruginosa* were diluted to 1 × 10^6^ CFU/mL with CAMHB, and 50 µL of bacterial suspension was loaded into 96-well plates. The final concentration of peptide was 1/4 MIC. Plates were covered and sealed following incubation at 37℃ for 24 h without agitation. An OD_600_ value increase of less than 0.005 was defined as no bacterial growth. The assay was performed in triplicate, with each sample performed in triplicate wells.

### Quantitative real-time PCR

An overnight culture of *P. aeruginosa* was diluted in CAMHB to 1 × 10^6^ CFU/mL, and a 50 µL aliquot was inoculated into a 96-well plate. Fifty microliters of LL-37 or RP557 at half MIC was added to each well. After incubation for 24 h, total RNA was extracted using the RNeasy Protect Bacteria Mini Kit (Qiagen, Hilden, Germany), and quality was assessed by the ratio of A260/A280 and A260/A230. The complementary DNA was synthesized using a High-Capacity cDNA Reverse Transcript Kit (Applied Biosystems, Lithuania, UAB). All samples were stored at −80°C until further analysis. The expression level of genes related to biofilm formation was assessed by qPCR using Rotor-Gene Q (Qiagen, Hilden, Germany). The 20 µL reaction system consisted of 10 µL EzAmp qPCR 2× master mix (SYBR Green, Low Rox, Daejeon, South Korea), 2 µg of cDNA, 10 pmol of forward primer, 10 pmol of reverse primer, and ultrapure water (MilliQ, Darmstadt, Germany) to a total volume of 20 µL. The cycling conditions of qPCR were as follows: initial denaturation at 94°C for 3 min, followed by 40 cycles of 15 s at 94°C, 25 s at 56°C, and 30 s at 72°C. The melting curve was obtained from 60°C to 95°C. The housekeeping gene *rpoD* was used for normalization. The relative changes in expression level were calculated using the 2^-△△ct^ method. The primers used in this study are listed in Table 1.

**TABLE 1:** Primers used in this study

Gene	Gene description		Forward (5′−3′)	Reverse (5′−3′)	Reference
*lasI*	Acyl-homoserine-lactone synthase	Quorum sensing system *las*	CTACAGCCTGCAGAACGACA	ATCTGGGTCTTGGCATTGAG	(18)
*lasR*	Transcriptional regulator LasR		ACGCTCAAGTGGAAAATTGG	GTAGATGGACGGTTCCCAGA	(18)
*lasA*	Protease LasA	Virulence factor	CTGCTGGCTTTCAAGGTTTC	CCAGCAAGACGAAGAGGAAC	(19)
*rhlA*	Rhamnosyltransferase subunit A	Rhamnolipid synthesis	AGCTGGGACGAATACACCAC	GACTCCAGGTCGAGGAAATG	(19)
*rhlB*	Rhamnosyltransferase subunit B		GAGCGACGAACTGACCTACC	CGTACTTCTCGTGAGCGATG	(19)
*rhlI*	Acyl-homoserine-lactone synthase	Quorum sensing system *rhl*	AAACCCGCTACATCGTCGC	TCTCGCCCTTGACCTTCTGC	(20)
*pslB*	Sugar-nucleotide production protein PslB	Exopolysaccharide *psl* synthesis	CAACGAATCCACCTTCATCC	ACTCGCCGCTCTGTACCTC	(20)
*pslD*	Outer membrane protein/secretion protein PslD		CGCTATCACGCCTACTTCCT	GACTTGGGCACGAAGACGAT	This study
*pelB*	Outer membrane protein/secretion protein PelB	Exopolysaccharide *pel* synthesis	AGCGCTTGCAACAGATTCTC	AACAGGTTCCAGTGGGTTTC	This study
*algA*	Sugar-nucleotide production AlgA	Exopolysaccharide alginate synthesis	CCATGATGATCGCCCACAAG	ACCTCGCAGTGGTTCTGGGT	(21)
*alg44*	c-di-GMP binding Alg44		CCACCAGATGAAAGGGACC	AAGATCACCTGGCCCTTGTT	This study
*algD*	GDP-mannose 6-dehydrogenase AlgD		CGCCGAGATGATCAAGTACA	AGGTTGAGCTTGTGGTCCTG	(22)
*algE*	Alginate production protein AlgE		GTGGCAGGACACCAACATC	GTGCGGTATTCGCTGAAACG	This study
*algK*	Outer membrane protein/secretion protein AlgK		CTCAAGCGCGAACAACAGAG	AACGGGAGCTGTTCATAGGC	This study
*rpoD*	RNA polymerase sigma factor RpoD (housekeeping gene)	Housekeeping gene	GCGACGGTATTCGAACTTGT	CGAAGAAGGAAATGGTCGAG	(22)

### Confocal laser scanning microscopy analysis

*Pseudomonas aeruginosa* overnight cultures were diluted to 1 × 10^6^ CFU/mL. An aliquot of 125 µL of bacterial suspension was cultured with 125 µL of LL-37 and RP557 solution at a quarter MIC in an eight-well chambered glass (LAB TEK, USA). The chambers were cultured at 37°C for 48 h to form mature biofilms. After incubation, the bacterial suspension was discarded, and the biofilm was washed with 0.85% saline. Biofilm was stained with the FilmTracer LIVE/DEAD Biofilm Viability Kit (Life Technologies Corporation, Eugene, USA) for 20 min in the dark. Then, the dye was discarded, and each well was rinsed. Biofilms were examined by confocal laser scanning microscopy on ZEISS LSM 800 (ZEISS, Germany) using a 20× objective. Laser excitation was performed at 488 nm for SYTO9 and 561 nm for PI. Emission was collected from 510 nm to 550 nm for SYTO 9 and 630 nm to 670 nm for PI. Cell viability was determined by the intensity of red and green fluorescence using ImageJ (NIH, MD, USA).

### Statistical analysis

The data were analyzed using one-way analysis of variance (ANOVA) and Dunnett’s T3 method by GraphPad Prism 9.4. The data are presented as the mean ± SD, and significance was established at *P* < 0.05.

## RESULTS

### Minimal inhibitory concentration of carbapenems, LL-37, and RP557 against carbapenem-resistant *P. aeruginosa*

The minimal inhibitory concentrations of carbapenems and antimicrobial peptides were determined by the microtiter dilution method. Except for the reference strain ATCC 27853, all strains were resistant to carbapenems. Among the carbapenem-resistant *P. aeruginosa* strains, NCCP 16079 isolated from human patients was extremely resistant to carbapenems, with MICs against ertapenem and meropenem greater than 2,048 μg/mL. For other strains isolated from companion dogs, the MIC against carbapenems was 16 times larger than the resistance criteria (Table 2). Peptide LL-37 was able to inhibit ATCC 27853 and other CRPA growth at 256 µg/mL (0.0570 µmol/mL), and RP557 was able to inhibit the reference strain and most CRPA at 32 µg/mL (0.0140 µmol/mL).

**TABLE 2:** MIC (μg/mL) of carbapenem-resistant *Pseudomonas aeruginosa*

*Pseudomonas aeruginosa* strain	MIC (μg/mL), [μmol/mL]				
	Ertapenem	Imipenem	Meropenem	LL-37	RP557
ATCC 27853	4 [0.0084]	4 [0.0134]	0.5 [0.0013]	256 [0.0570]	32 [0.0140]
NCCP 16079	>2,048 [4.3069]	256 [0.8551]	>2,048 [5.3408]	256 [0.0570]	32 [0.0140]
D7	128 [0.2692]	64 [0.2138]	32 [0.0834]	256 [0.0570]	32 [0.0140]
D16	128 [0.2692]	64 [0.2138]	32 [0.0834]	256 [0.0570]	32 [0.0140]
D25	128 [0.2692]	256 [0.8551]	32 [0.0834]	256 [0.0570]	32 [0.0140]
D26	128 [0.2692]	64 [0.2138]	64 [0.1669]	256 [0.0570]	32 [0.0140]
B4	128 [0.2692]	128 [0.4276]	32 [0.0834]	256 [0.0570]	32 [0.0140]

### Biofilm inhibition effect of LL-37 and RP557

**FIG 1:**
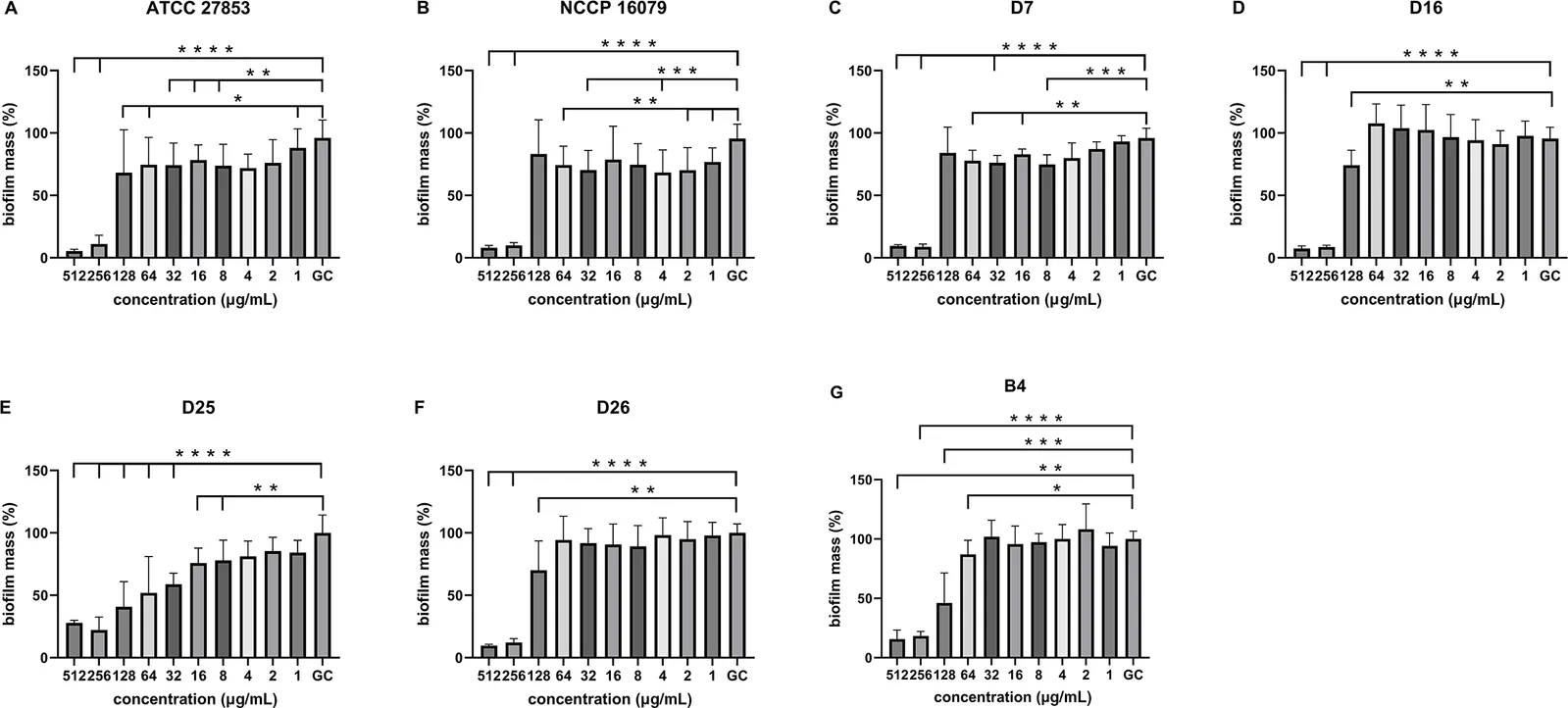
Inhibitory effect of LL-37 on carbapenem-resistant *Pseudomonas aeruginosa* biofilm formation. *, *P* < 0.05; * *, *P* < 0.01; * * *, *P* < 0.001; * * * *, *P* < 0.0001.

**FIG 2:**
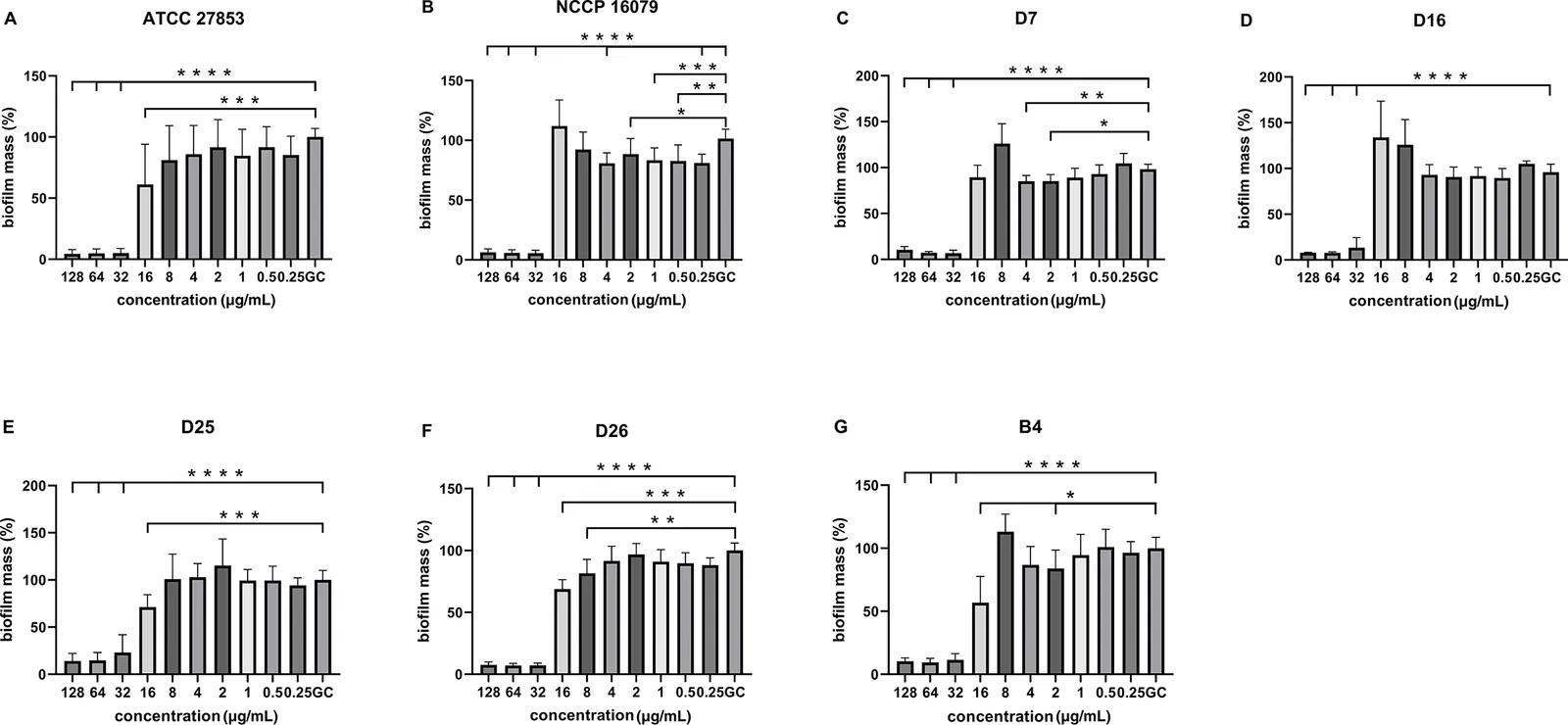
Inhibitory effect of RP557 on carbapenem-resistant *Pseudomonas aeruginosa* biofilm formation. *, *P* < 0.05; * *,: *P* < 0.01; * * *, *P* < 0.001; * * * *, *P* < 0.0001.

**FIG 3:**
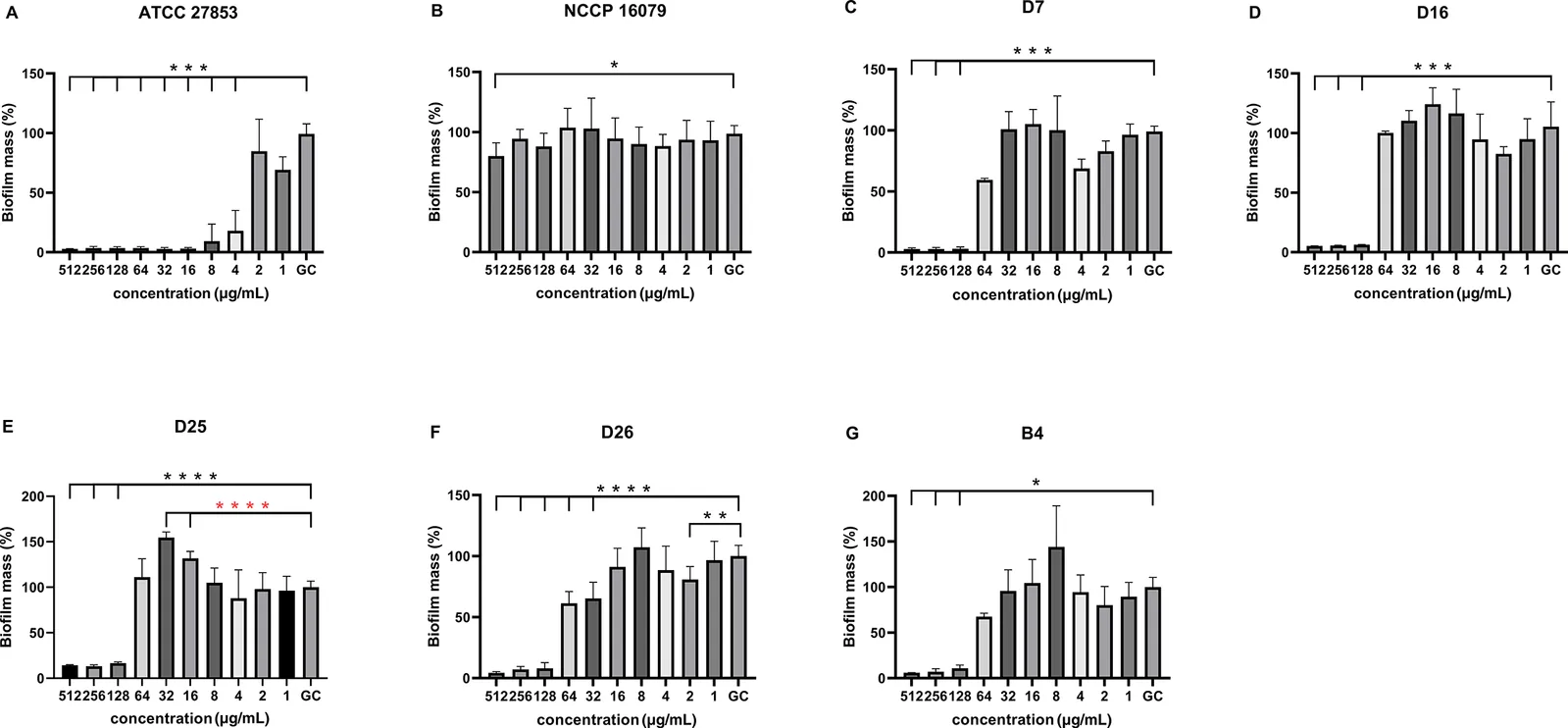
Effect of ertapenem on carbapenem-resistant *Pseudomonas aeruginosa* biofilm. Asterisks in black represent significant decrease, and asterisks in red represent significant increase. *, *P* < 0.05; * *, *P* < 0.01; * * *, *P* < 0.001; * * * *, *P* < 0.0001.

**FIG 4:**
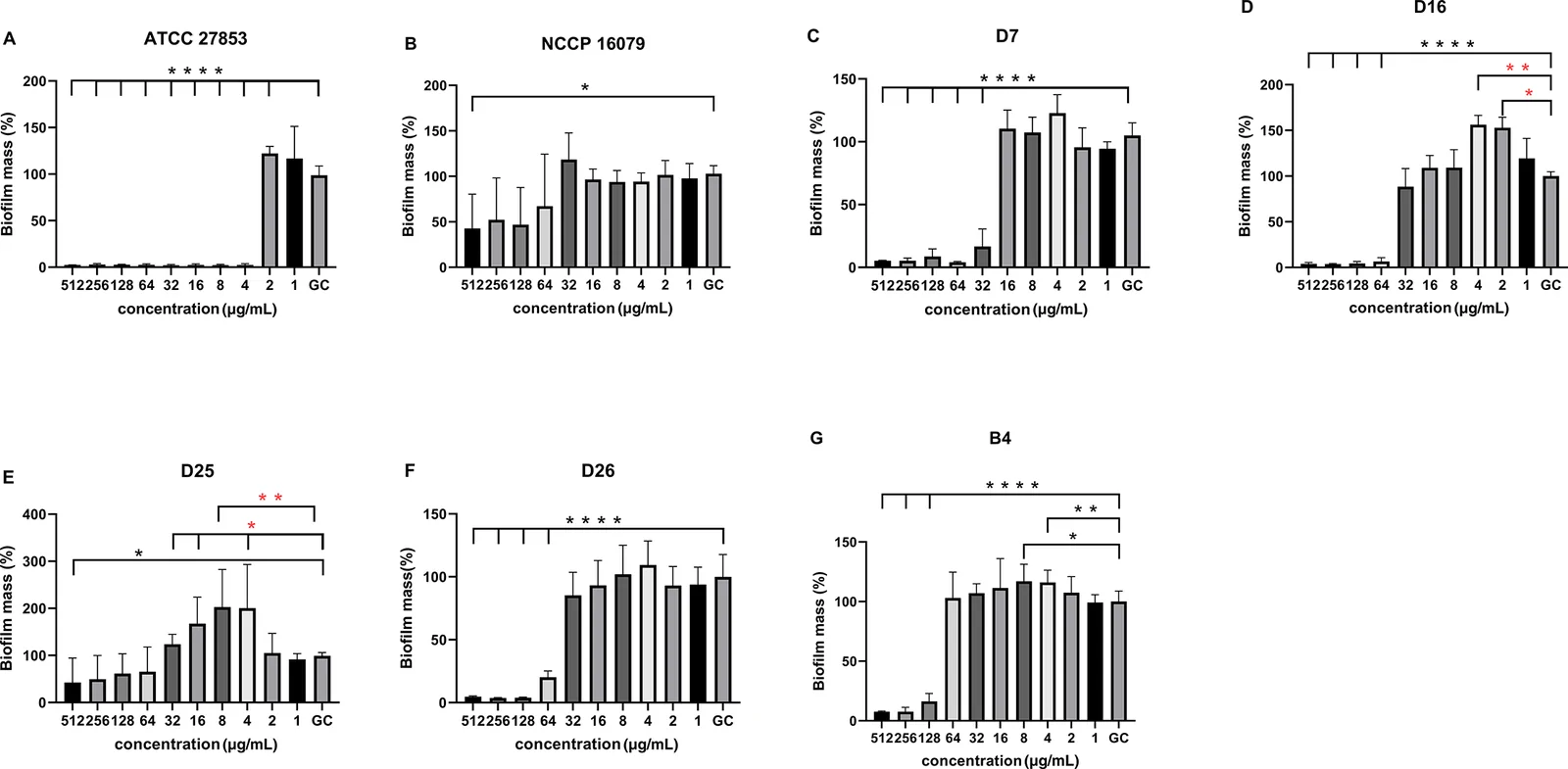
Effect of imipenem on carbapenem-resistant *Pseudomonas aeruginosa* biofilm. Asterisks in black represent significant decrease, and asterisks in red represent significant increase. *, *P* < 0.05; * *, *P* < 0.01; * * *, *P* < 0.001; * * * *, *P* < 0.0001.

**FIG 5:**
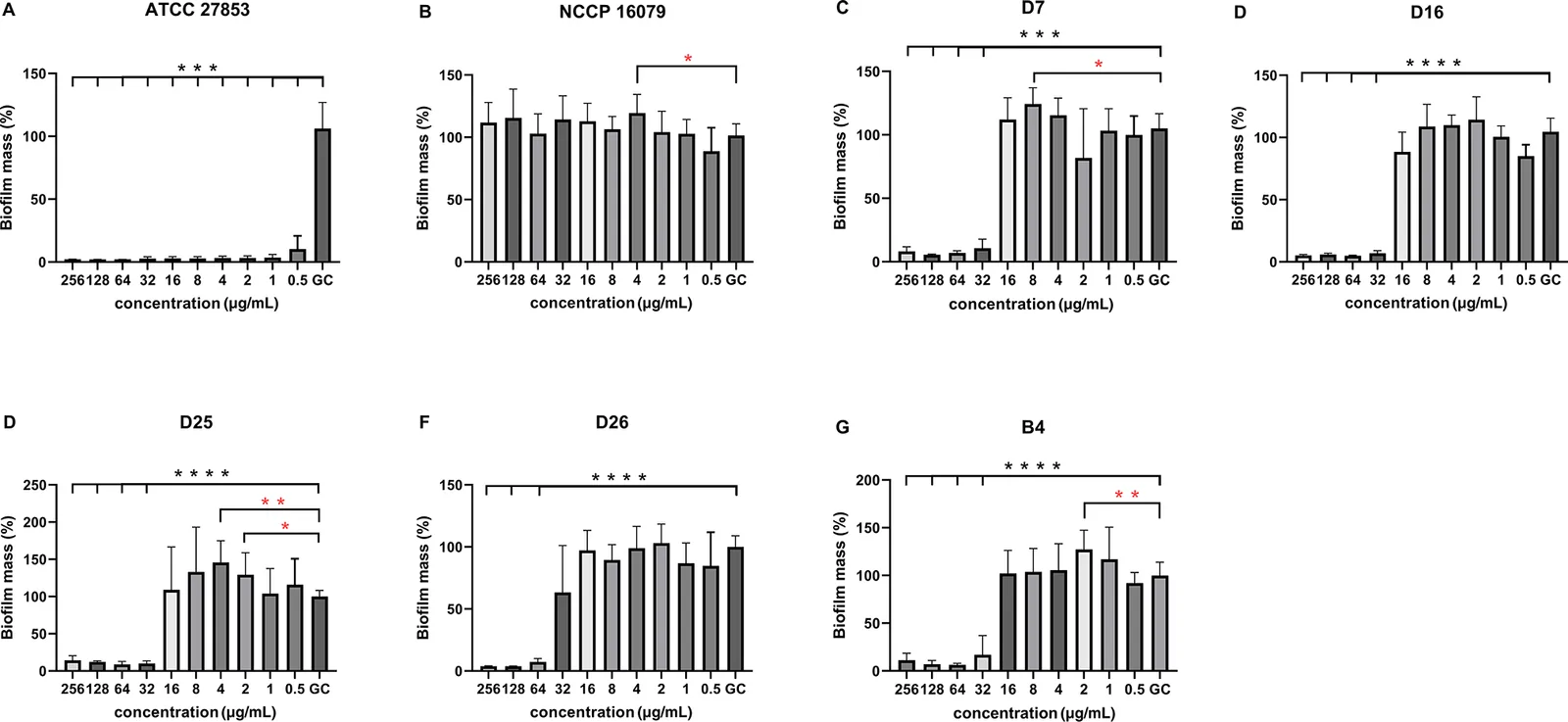
Effect of meropenem on carbapenem-resistant *Pseudomonas aeruginosa* biofilm. Asterisks in black represent significant decrease, and asterisks in red represent significant increase. *, *P* < 0.05; * *,: *P* < 0.01; * * *, *P* < 0.001; * * * *, *P* < 0.0001.

Concentrations ranging from 512 μg/mL to 1 μg/mL of LL-37 and from 128 μg/mL to 0.25 μg/mL were used to assess the biofilm inhibition effect. LL-37 has a moderate inhibition effect on biofilm formation of all seven strains at a concentration lower than the MIC (Fig. 1). The inhibition effect was most distinct when treating D25 and B4. For RP557, biofilm formation of six strains was partly inhibited at subMICs, except for D16, in which biofilm formation was inhibited at an MIC of 32 µg/mL (0.0140 µmol/mL) (Fig. 2). The inhibition effect was most distinct when treating B4. The MBIC of LL-37 and RP557 equals the MIC, except for D25 and B4. However, in the groups treated with carbapenems, low concentrations of carbapenems caused significant increases in biofilm mass (Fig. 3 to 5). The MBIC of ertapenem equals the MIC, except for strain D25 and 16079. Similar results were found in imipenem for D25 and B7, as well as meropenem for B7.

### Biofilm removal effect of LL-37 and RP557

**FIG 6:**
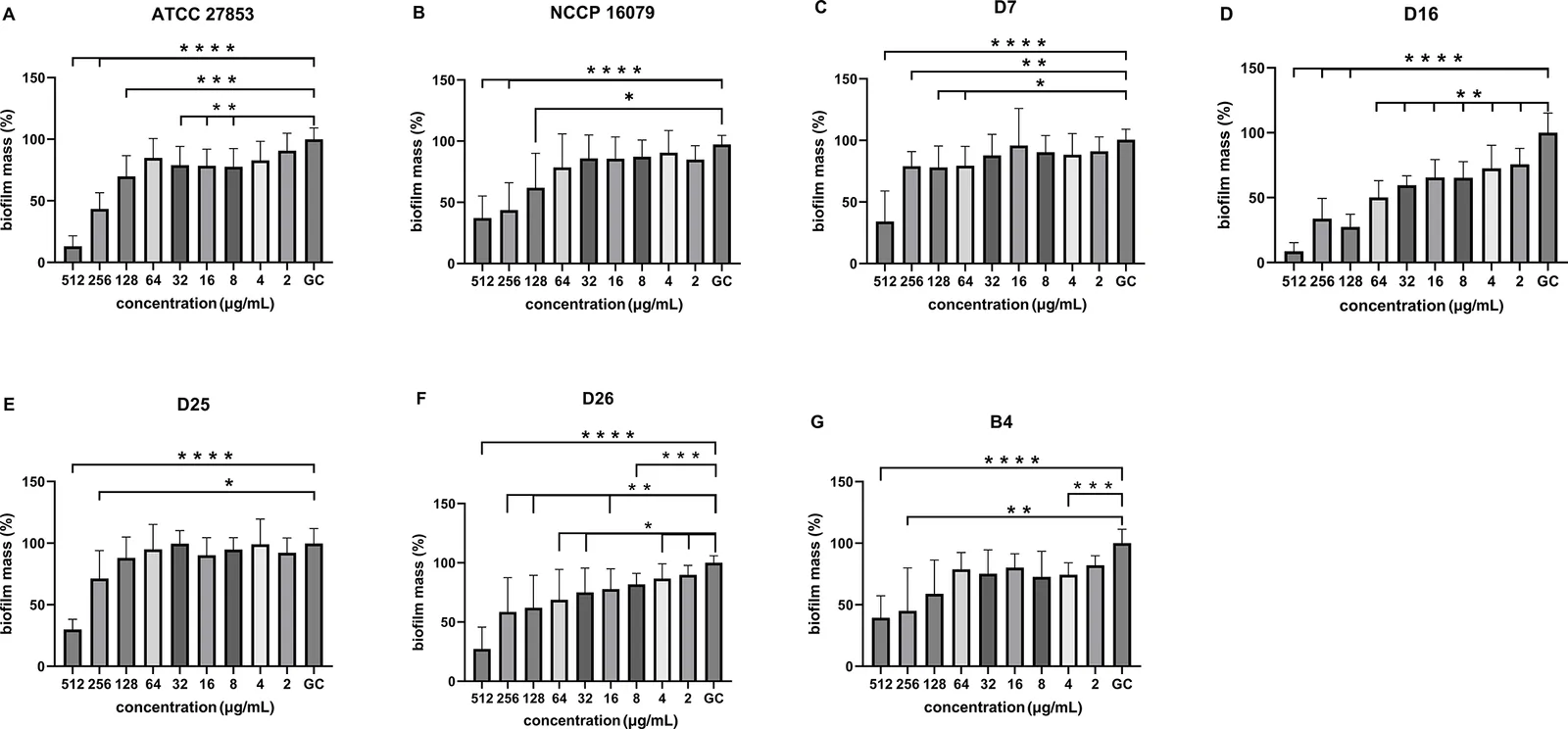
Biofilm removing effect of LL-37. *,: *P* < 0.05; * *,: *P* < 0.01; * * *, *P* < 0.001; * * * *, *P* < 0.0001.

**FIG 7:**
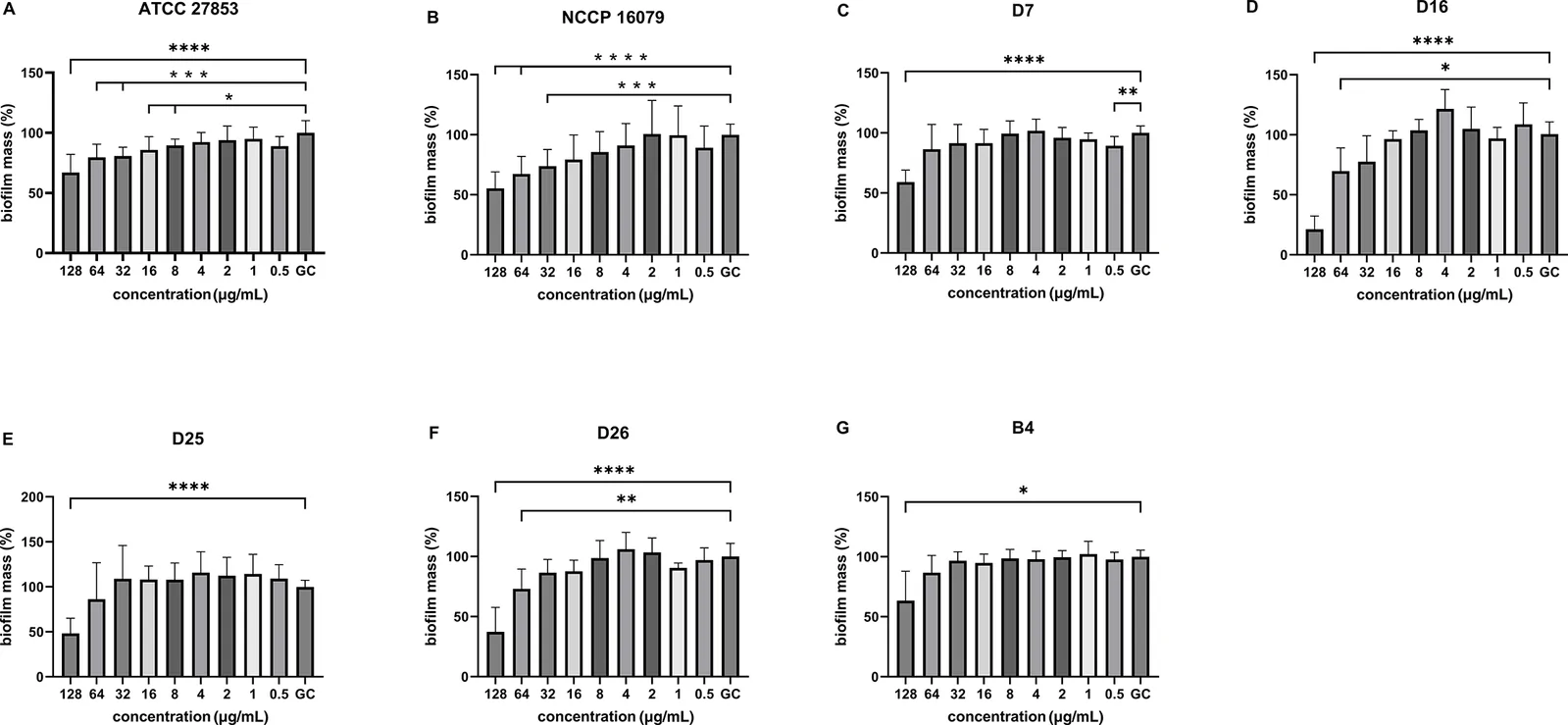
Biofilm removing effect of RP557. *, *P* < 0.05; * *, *P* < 0.01; * * *, *P* < 0.001; * * * *, *P* < 0.0001.

Concentrations ranging from 512 μg/mL to 1 μg/mL and from 128 μg/mL to 0.5 μg/mL were used to assess its effect on mature *P. aeruginosa* biofilm of LL-37 and RP557, respectively. LL-37 removed 25–70% of the mature biofilm of the *P. aeruginosa* strains used in this study at 256 µg/mL (0.0570 µmol/mL). For strains ATCC 27853, NCCP 16079, D16, and D26, LL-37 was even able to remove part of the mature biofilm at a concentration lower than its MIC of 256 µg/mL (Fig. 6). For all seven strains, RP557 removed part of mature biofilms at concentrations of 2–4 times the MIC, and was more effective in treating D16, D25, and D26, with more than 50% of mature biofilm removed (Fig. 7). No MBEC was detected for both LL-37 and RP557.

### Synergetic effect of RP557 and LL-37 with carbapenems on carbapenem-resistant *P. aeruginosa*

A quarter MIC of LL-37 (64 µg/mL, 0.0142 µmol/mL) and RP557 (8 µg/mL, 0.0035 µmol/mL) was used together with carbapenems to assess the combined effect of the two antimicrobial peptides with carbapenems. Coadministration of LL-37 at subMICs and carbapenems increased the susceptibility of CRPA to carbapenems by 4- to 32-fold (Table 3). The combination of subMIC RP557 and carbapenems had a synergetic effect on inhibiting CRPA growth (Table 4). When 8 µg/mL RP557 was used together, the susceptibility of CRPA to carbapenems increased by 4- to 16-fold.

**TABLE 3:** Synergetic effect of subMICs LL-37 and carbapenems against carbapenem-resistant *Pseudomonas aeruginosa*

*Pseudomonas aeruginosa* strain	MIC (μg/mL) of combined use with LL-37 (initial MIC)		
	Ertapenem	Imipenem	Meropenem
ATCC 27853	4 (4)	1 (4)	0.5 (0.5)
NCCP 16079	>256 (>2048)	64 (256)	>256 (>2048)
D7	16 (128)	8 (64)	8 (32)
D16	16 (128)	8 (64)	8 (32)
D25	16 (128)	8 (256)	4 (32)
D26	16 (128)	16 (64)	4 (64)
B4	16 (128)	8 (128)	4 (32)

**TABLE 4:** Synergetic effect of subMICs RP557 and carbapenems against carbapenem-resistant *Pseudomonas aeruginosa*

*Pseudomonas aeruginosa* strain	MIC (μg/mL) of combined use with RP557 (initial MIC)		
	Ertapenem	Imipenem	Meropenem
ATCC 27853	2 (4)	1 (4)	0.5 (0.5)
NCCP 16079	>256 (>2,048)	32 (256)	>256 (>2,048)
D7	16 (128)	16 (64)	8 (32)
D16	16 (128)	16 (64)	8 (32)
D25	16 (256)	16 (256)	4 (32)
D26	16 (256)	16 (64)	4 (64)
B4	16 (128)	16 (128)	8 (32)

### Biofilm-related gene expression level changed in *P. aeruginosa* treated with LL-37 and RP557

**FIG 8:**
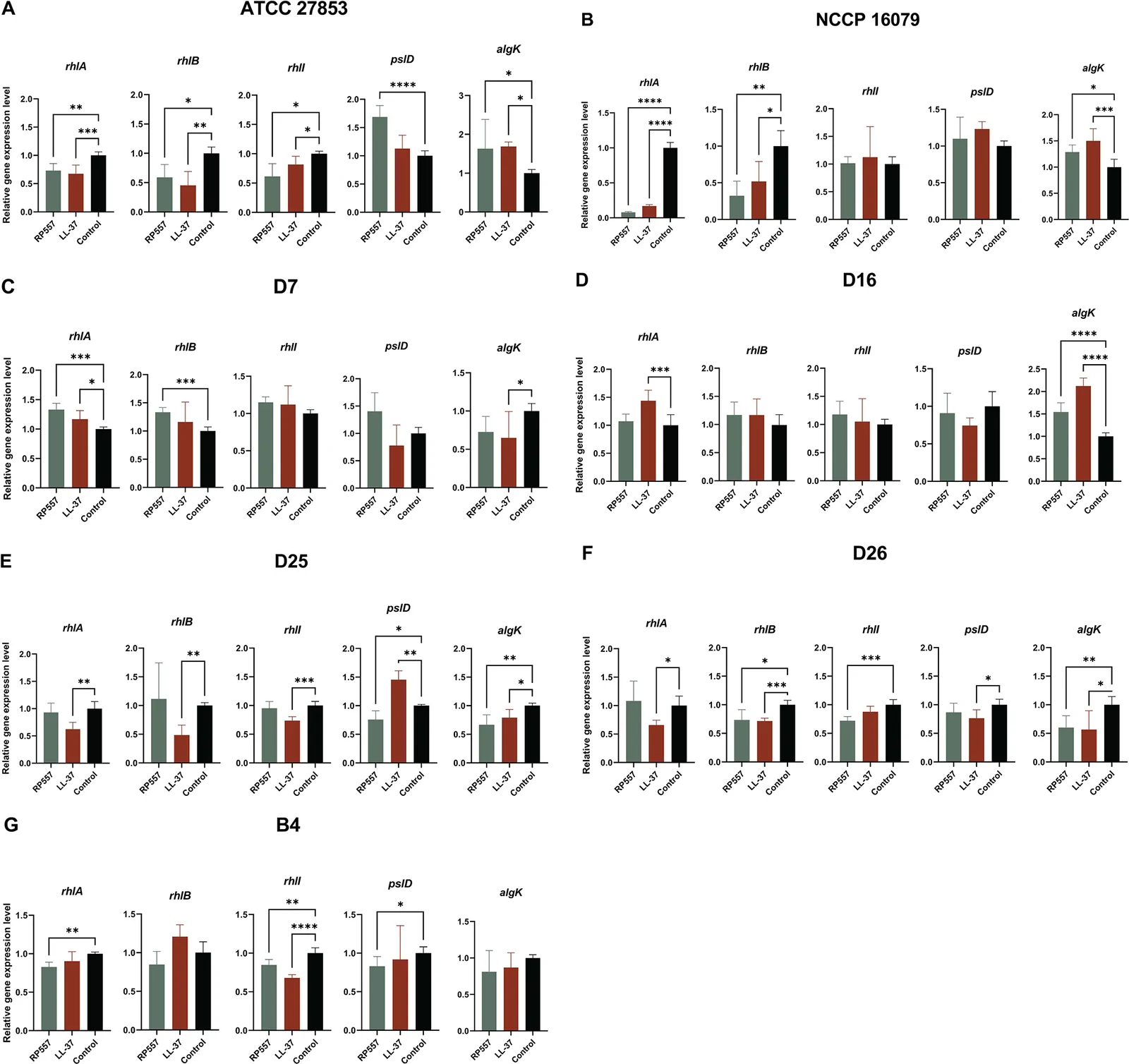
The effects of LL-37 and RP557 on biofilm formation-related genes. *, *P* < 0.05; * *, *P* < 0.01; * * *, *P* < 0.001; * * * *, *P* < 0.0001.

Fourteen genes related to the quorum sensing system and biofilm information were selected for gene expression level analysis by qPCR. Among the 14 genes selected, five genes had significant expression differences in the LL-37- or RP557-treated groups (Fig. 8). Within the total of seven strains, five strains treated with antimicrobial peptides showed significant downregulation of *rhlA* and *rhlB* expression, and two strains showed upregulation of *rhlA* or *rhlB* in the treated groups. *rhlI* expression was significantly suppressed in the treatment groups except for strains NCCP 16079, D7, and D16. For *pslD*, its expression was significantly upregulated in RP557-treated ATCC 27853 and LL-37-treated D25. However, there was a significant decrease in *pslD* expression in D25 and B4 treated with RP557 and D26 treated with LL-37. For *algK*, its expression in ATCC 27853, NCCP 16079, and D16 treated by antimicrobial peptides was induced, while in treated D7, D25, and D26, its expression was repressed.

### The biofilm structure and cell viability changed after treatment with LL-37 or RP557

**Fig 9:**
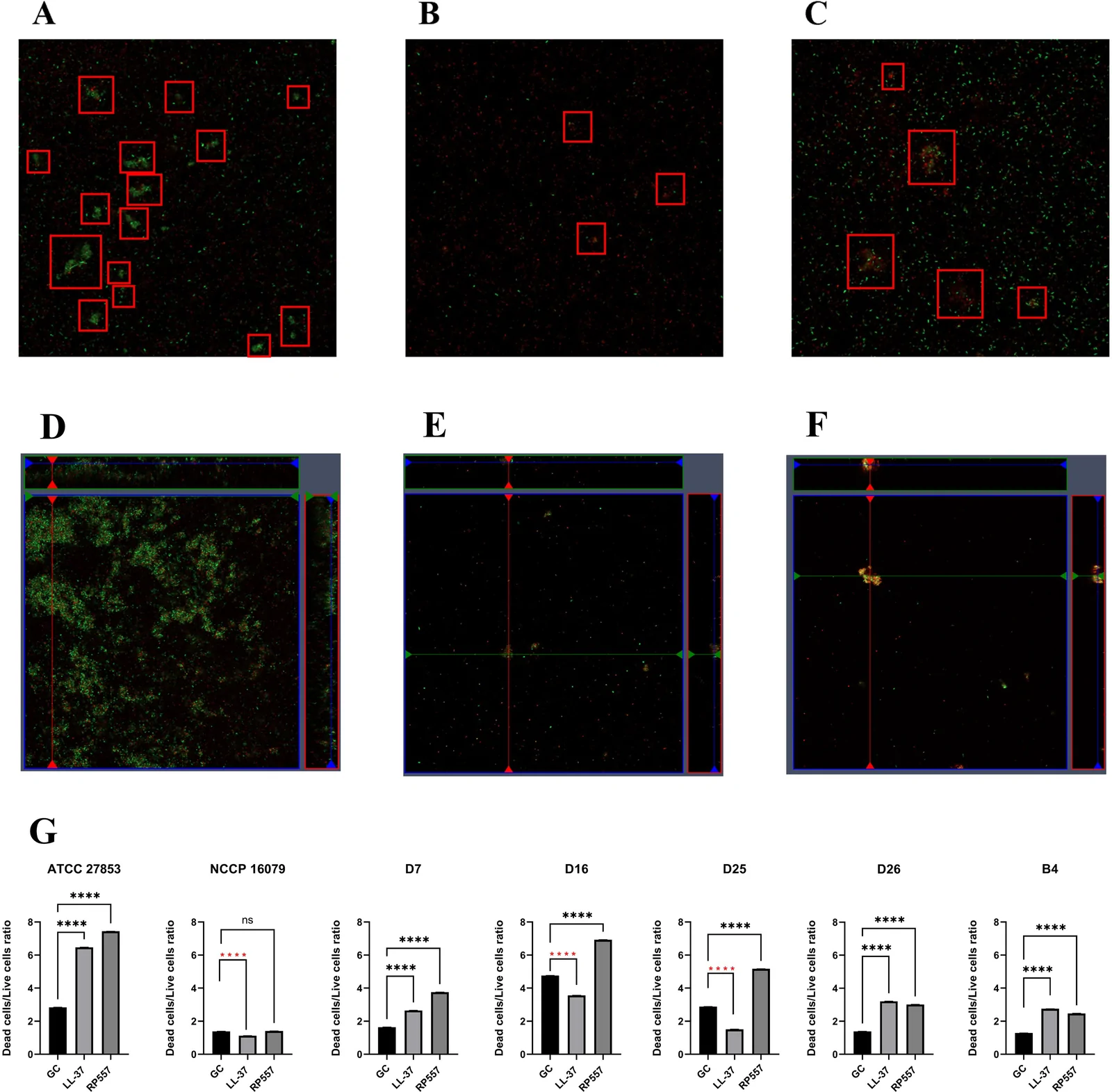
Confocal laser scanning microscopy images after staining showing the biofilm structure of *P. aeruginosa*. Orthogonal views were in the same *Z*-position. (**A**) Confocal images of ATCC 27853 without LL-37 or RP557 treatment. (**B**) Confocal images of ATCC 27853 taken at 48 h with LL-37 treatment. (**C**) Confocal images of ATCC 27853 taken at 48 h with RP557 treatment. (D) Orthogonal view of ATCC 27853 without treatment. (E) Orthogonal view of ATCC 27853 with LL-37 treatment. (F) Orthogonal view of RP557 treatment. (G) Biofilm cell viability of *P. aeruginosa* in different-treated groups. The cell viability was represented by the ratio of red and green fluorescence intensity. Asterisks in black represent significant decrease in cell viability, and asterisks in red represent significant increase in cell viability. *, *P* < 0.05; * *, *P* < 0.01; * * *, *P* < 0.001; * * * *, *P* < 0.0001.

The biofilm structure was visualized by confocal laser scanning microscopy, and the biofilm cell viability was assessed by comparing fluorescence intensity (Fig. 9). The live cells were stained green by SYTO 9 and the dead cells were stained red by propidium iodide (PI). The mature biofilm structures were significantly reduced in the LL-37- and RP557-treated groups (see Fig. S1 in the supplementary material). In addition, the cell viability of the RP557-treated group was significantly decreased in all seven strains, and four strains treated with LL-37 had lower viability than the control group.

## DISCUSSION

*Pseudomonas aeruginosa* has received a lot of concern due to its biofilm-forming ability and increasing antimicrobial resistance (2). Both biofilm-forming ability and antimicrobial resistance make *P. aeruginosa* infection hard to cure. As the most effective β-lactam antibiotic, carbapenems are used to treat severe ESBL-producing bacteria infections, but since carbapenem resistance was first found in the 1980s, carbapenem-resistant *P. aeruginosa* infections have been reported widely, and now it has been listed as a critical pathogen together with carbapenem-resistant Enterobacteriaceae and carbapenem-resistant *Acinetobacter baumannii* (23). Almost 300 cases of CRPA infection in 274 patients have been reported, and most of the infected patients were hospitalized long-term (24). Therefore, new antimicrobial compounds need to be developed to combat CRPA infections.

In this study, a new synthetic peptide, RP557, was selected for assessing its antimicrobial and antibiofilm effects. Derived from human cathelicidin LL-37, RP557 had a similar antimicrobial and antibiofilm effect against the *P. aeruginosa* reference strain (25). Our results were also consistent with the previous study of the effect of RP557 on several biofilm-forming bacteria, including *Pseudomonas aeruginosa*, *Klebsiella pneumoniae*, Methicillin-resistant *Staphylococcus aureus,* and *Mycobacterium abscessus* (13, 14). Furthermore, to our knowledge, there are no previous studies that have revealed the effect of LL-37 and RP557 on CRPA. Therefore, our study provides a potential way of dealing with carbapenem-resistant *P. aeruginosa*.

The antimicrobial effect of LL-37 has been reported previously. However, compared to its antimicrobial effect, its antibiofilm effect is more important since it can inhibit biofilm formation at very low concentrations (26). Our results show that LL-37 at 256 µg/mL (0.0570 µmol/mL) inhibits the growth of both *P. aeruginosa* reference and carbapenem-resistant strains. In addition to inhibiting planktonic cell growth, it inhibited the biofilm information at subMIC. As for RP557, it showed a better bacterial inhibition effect because its MIC for both *P. aeruginosa* reference and CPRA strains was 32 µg/mL (0.0140 µmol/mL), which is one-eighth of the LL-37 MIC. Our results are consistent with those previously reported, in which both LL-37 and RP557 can inhibit *P. aeruginosa* growth regardless of resistance to other antibiotics, although the MIC of LL-37 and RP557 against reference strains are different from those previously reported (13). Thus, it is speculated that the difference during the synthesis process is responsible for the difference in MIC. RP557 also has an antibiofilm effect at concentrations lower than the MIC. The effective concentration of antibiofilm varies among strains, but most of them are effective at one-eighth to half MIC, which is from 4 μg/mL to 16 μg/mL (0.0018 to 0.0070 µmol/mL). There was an interesting result observed as well. For strains D25 and B4, the biofilm inhibition effect of both peptides is less effective than that of other strains when treating in MIC. However, in concentrations lower than MIC, the biofilm inhibition effect was better when compared to treating other strains. We suppose the difference may be related to the different strain phenotype or origin, as D25 was isolated from pyoderma and B4 was isolated from feces, while others were isolated from otitis. The effective concentration of RP557 in the current study is also safe to use, as it didn’t represent cytotoxicity to mammalian cells even at a high concentration of 256 µg/mL (27). In contrast, biofilm mass in CPRA treated with a low concentration of carbapenems was significantly increased, indicating that it may induce biofilm formation, increasing its resistance against carbapenems.

Mature biofilm is usually more resistant to drugs because there are many components in the biofilm matrix, including eDNA, exopolysaccharides, and proteins. The penetration of some antimicrobial compounds can be delayed by biofilm matrices, especially in *P. aeruginosa* mucoid strain in which the biofilm contains a large amount of alginate (3). In addition, the existence of anionic eDNA will reduce the antimicrobial activity of cationic antimicrobial peptides such as polymyxin B. Additionally, the persister cells in the mushroom-like structure of biofilm are not susceptible to antibiotics that act in the growth phase, such as β-lactam antibiotics, making the biofilm more resistant and hard to eliminate (16). Thus, the effects of LL-37 and RP557 on mature biofilm have been assessed. We found that 256 µg/mL of LL-37 removed approximately 30–70% of the mature biofilm, and LL-37 was also able to remove mature biofilm of some strains at subMIC. RP557 reduced approximately 40–80% of mature biofilm at 128 µg/mL which is four times the MIC. In our study, LL-37 showed better biofilm removal ability than RP557 compared to the MIC. LL-37 has been reported to be effective in eradicating *P. aeruginosa* biofilm at subMIC, which is consistent with our results (11, 28). In the previous study, RP557 was able to kill 50% of *P. aeruginosa* biofilm cells at a concentration of 32 µg/mL, and 90% of biofilm cells at a concentration of approximately 100 µg/mL (13).

Since biofilms contribute a lot to antibiotic resistance, LL-37 and RP557 in subinhibitory concentrations were coadministered with carbapenems to investigate their combined effect (29). The results showed that coadministration of LL-37 or RP557 with carbapenems resulted in a significant increase in the susceptibility of CRPA to carbapenems, with MIC reduced by 4–16 times compared to its initial MIC. The synergetic effect of the antimicrobial peptide with other antibiotics has been verified in other studies (28, 30). It suggests that the carbapenem resistance of CRPA may be associated with its biofilm. However, strain NCCP 16079 maintained a high level of carbapenem resistance when coadministered with LL-37 or RP557, suggesting that the carbapenem resistance of NCCP 16079 is more likely associated with other resistance mechanisms, such as the carbapenemase it produced.

To further investigate the effect of LL-37 and RP557 on CRPA biofilms, 14 genes related to biofilm formation were selected for qPCR analysis. Among the 14 genes tested, 5 genes showed significant expression level changes when treated with LL-37 or RP557 at a quarter MIC. *rhlA* and *rhlB* were repressed in ATCC 27853, D25, D26, and B4. *rhlA* and *rhlB* are the genes essential for rhamnolipid synthesis in *P. aeruginosa*. Rhamnolipid, a kind of biosurfactant produced by *P. aeruginosa*, is not only one of the main virulence factors of *P. aeruginosa*, but it also has an important role in maintaining the typical mushroom-like structure of *P. aeruginosa* biofilms and inducing the dispersion of biofilm (31). Downregulation of *rhlA* and *rhlB* results in a decrease in the swarming ability of *P. aeruginosa*, which is essential for the initial attachment of *P. aeruginosa* to the surface, thus influencing biofilm formation (32). This result was consistent with the results of the biofilm inhibition assay, which showed that at a quarter MIC, the biofilm mass of these downregulated strains was significantly less than that of the untreated group. Additionally, the repressed production of rhamnolipid will lead to the formation of a flat biofilm rather than a mushroom-shaped structure (33), which is the same as the results presented in conlocal laser scanning microscopy images. This could be the reason for the synergistic effect of LL-37 and RP557 with carbapenem since the persister cells inside the mushroom structure contribute a lot to antimicrobial resistance, especially in chronic infection (4).

*rhlI* expression level was reduced in ATCC 27853, D25, D26, and B4. RHLI is responsible for synthesizing *N*-butyryl-L-homoserine lactone, the cognate AHL of RhlR, together with RhlR forming the RHL QS system (34). The RHL system regulates biofilm formation by regulating exopolysaccharide production by regulating the *pel* genes (35). Reduction in pel polysaccharides affects pellicle formation and biofilm structure (36). The fold changes in expression of *pslD,* and *algK* differed in different strains. PslD is the secretion protein in exopolysaccharide synthesis, thus involving biofilm formation, and AlgK is a secretion protein related to alginate synthesis (37). The changes in *pslD* and *algK* expression were also according to the results from the biofilm inhibition assay, as the biofilm mass of groups with upregulation of *pslD* and *algK* treated by a quarter MIC of LL-37 or RP557 was neither increased nor reduced. These results suggest that biofilm formation is a comprehensive process regulated by many genes, and changes in some genes may not affect overall biofilm matrix production.

To study the effect of LL-37 and RP557 on *P. aeruginosa* biofilm structure, confocal laser scanning microscopy images were taken. The confocal laser scanning microscopy images showed that the mushroom-like structure was reduced in LL-37- or RP557-treated groups. This result was consistent with the decreased expression level of *rhlA* and *rhlB as* rhamnolipid reduction could induce the biofilm to adapt a flat form rather than the typical mushroom structure. The monolayer was considered more sensitive to antibiotics (26), which could be the reason for the synergetic effect between RP557 and antibiotics observed in the present study. Compared to the nontreated group, the biofilm cell viability of *P. aeruginosa* in LL-37- or RP557-treated groups decreased as well. Peptides were reported to have the potential ability to kill biofilm cells (38 – 40). RP557 was proved effective in killing methicillin-resistant *Staphylococcus aureus* (MRSA), *P. aeruginosa*, multiple drug resistant (MDR) *S. epidermidis*, and *M. abscessus* biofilm cells in the previous study. Most peptides play their antimicrobial effect by disrupting bacteria’s cell membranes. Along with the biofilm inhibition ability, peptides are easier to penetrate into the bacteria’s membrane, thus increasing the killing efficiency.

In conclusion, our study presented the antimacrobial and antibiofilm effects of RP557 and its origin, LL-37, on carbapenem-resistant *P. aeruginosa* for the first time. Overall, RP557 had a better antimicrobial effect than LL-37. Both of the two peptides were able to inhibit biofilm formation and have a moderate effect on removing mature biofilm. We also presented the synergy between RP557 and carbapenems, and the result also showed that biofilm may be associated with antibiotic resistance. In addition, the mechanism of RP557 and LL-37 on biofilm inhibition may be by affecting the biofilm structure. However, there are still some obstacles that need to be overcome for antimicrobial peptide to be used as antibiotics. First and foremost, the high cost of synthesizing, purifying, and preserving peptides is hindering their widespread utilization. Instead of traditional synthetic methods, biosynthetic methods may help reduce the cost (41). Another important limitation is the potential cell toxicity. Due to its specific affinity to membrane structure, it can interact not only with bacterial cells but also with eukaryotic cells, leading to potential cytotoxicity. The membrane affinity results in a relatively low utilization ratio as well (25). Thus, a new trend is to combine AMPs into some delivery systems, such as PLGA and chitosan, which will help reduce the cytotoxicity and improve efficiency (42). Further work is needed to evaluate the efficacy of RP557 on other biofilm-forming carbapenem-resistant bacteria, such as carbapenem-resistant Enterobacteriaceae and carbapenem-resistant *A. baumannii* to see if the antibiofilm effect is universal or species-specific. It could also be useful to develop a nanoparticle-RP557 delivery system. In any case, RP557 may be a potential antimicrobial compound to treat carbapenem-resistant *P. aeruginosa* infections.

## SUPPLEMENTAL MATERIAL

Fig. S1 spectrum.00430-23-s0001.pdf Fig S1. Confocal laser scanning images of *P. aeruginosa* isolates. (A) Strain NCCP 16079. (B) Strain D7. (C) Strain D16. (D) Strain D25. (E) Strain D26. (F) Strain B4.

ASM does not own the copyrights to Supplemental Material that may be linked to, or accessed through, an article. The authors have granted ASM a non-exclusive, world-wide license to publish the Supplemental Material files. Please contact the corresponding author directly for reuse.
