# Supplementary material for: Effects of synthetic peptide RP557 and its origin, LL-37, on carbapenem-resistant Pseudomonas aeruginosa
Source: Microbiol Spectr. 2023 Aug 9;11(5):e00430-23. doi: 10.1128/spectrum.00430-23 (PMC10581083; doi:10.1128/spectrum.00430-23)
Supplement: Fig. S1 — Fig S1. Confocal laser scanning images of P. aeruginosa isolates. (A) Strain NCCP 16079. (B) Strain D7. (C) Strain D16. (D) Strain D25. (E) Strain D26. (F) Strain B4. [file spectrum.00430-23-s0001.pdf]

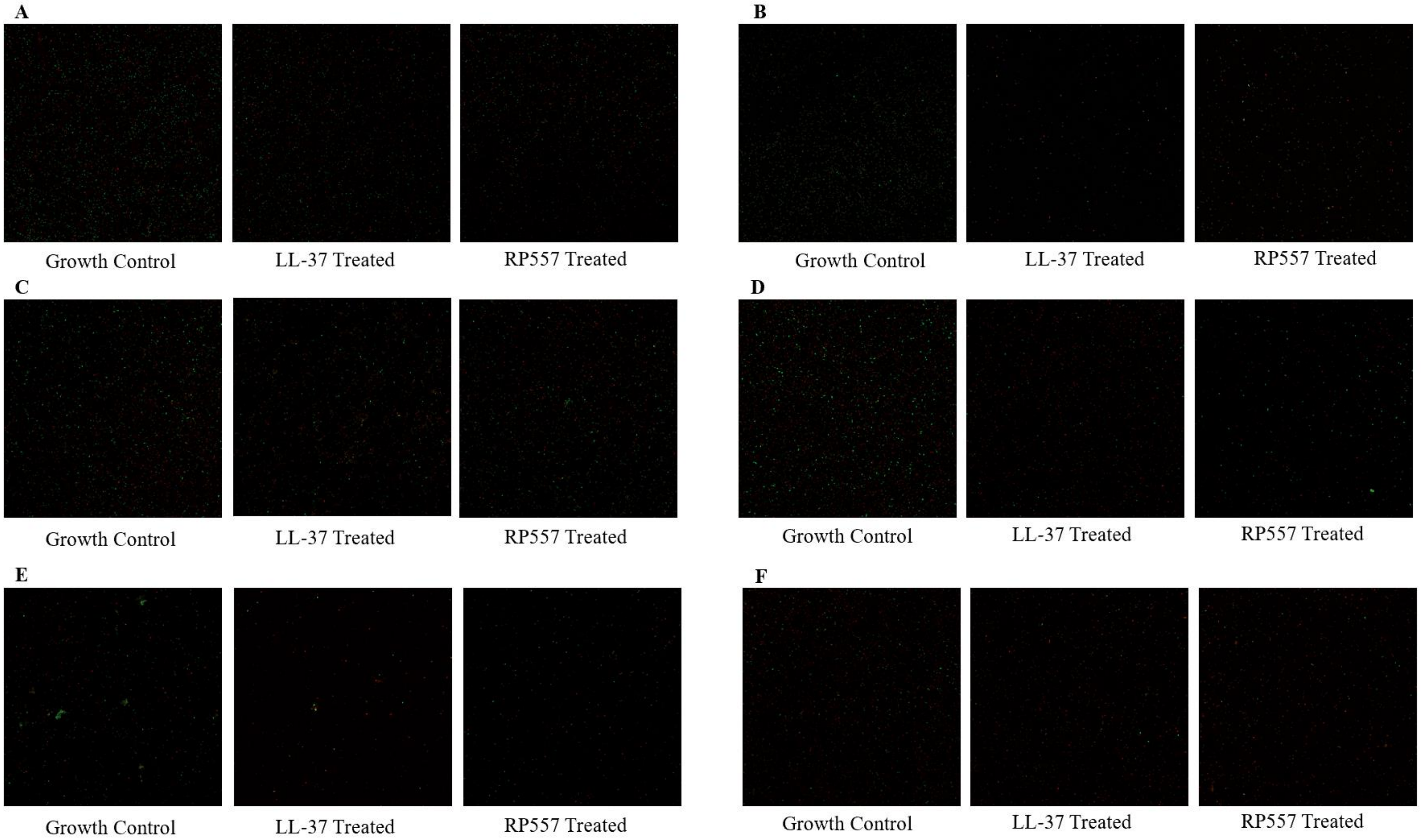

FIG S1 Confocal laser scanning images of *P. aeruginosa* isolates. (A) Strain NCCP 16079 (B) Strain D7 (C) Strain D16 (D) Strain D25 (E) Strain D26 (F) Strain B4
